# Large magnetocaloric effect and adiabatic demagnetization refrigeration with YbPt_2_Sn

**DOI:** 10.1038/ncomms9680

**Published:** 2015-10-23

**Authors:** Dongjin Jang, Thomas Gruner, Alexander Steppke, Keisuke Mitsumoto, Christoph Geibel, Manuel Brando

**Affiliations:** 1 Max Planck Institute for Chemical Physics of Solids, Noethnitzer Strasse 40, D-01187 Dresden, Germany; 2 Graduate School of Science and Technology, Niigata University, 8050, Ikarashi 2-nocho, Niigata 950-2181, Japan

## Abstract

Adiabatic demagnetization is currently gaining strong interest in searching for alternatives to ^3^He-based refrigeration techniques for achieving temperatures below 2 K. The main reasons for that are the recent shortage and high price of the rare helium isotope ^3^He. Here we report the discovery of a large magnetocaloric effect in the intermetallic compound YbPt_2_Sn, which allows adiabatic demagnetization cooling from 2 K down to 0.2 K. We demonstrate this with a home-made refrigerator. Other materials, for example, paramagnetic salts, are commonly used for the same purpose but none of them is metallic, a severe limitation for low-temperature applications. YbPt_2_Sn is a good metal with an extremely rare weak magnetic coupling between the Yb atoms, which prevents them from ordering above 0.25 K, leaving enough entropy free for use in adiabatic demagnetization cooling. The large volumetric entropy capacity of YbPt_2_Sn guarantees also a good cooling power.

## Abstract (web summary)

Magnetocaloric materials are used in adiabatic demagnetization refrigerators to reach extremely low temperature without using Helium. Here, the authors report a large magnetocaloric effect in YbPt2Sn, and show how the properties of this alloy makes it a good magnetocaloric material.

## Graphical abstract

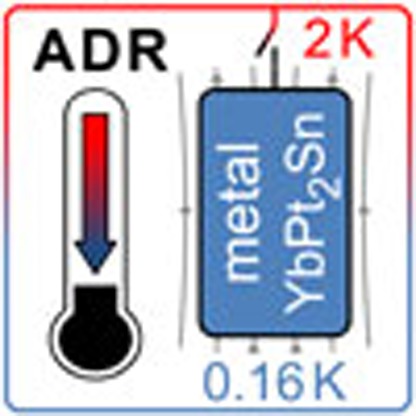

Usage of ^3^He is found in a variety of fields encompassing medical applications, military purpose and scientific research^1^,^2^,^3^. In science and engineering, ^3^He is one of the essential ingredients to achieve very low temperatures^4^ or to detect radioactivity^1^. We experience today a period of supply cutbacks for miscellaneous demands and very high costs for pure ^3^He: For example, low-temperature physicists have required only 1.3% of the available ^3^He between 2004 and 2010, and the price rose by a factor of 15 (refs 2, 3). Before this cutback, they were the major consumers of ^3^He, since temperatures down to 0.3 K and below were mostly reached by ^3^He systems and ^3^He/^4^He dilution refrigerators^4^. Given that ^3^He has been solely obtained as a byproduct from decay of tritium in nuclear weapons stockpiles or nuclear reactors, global agreement to reduce those facilities still holds, it seems unlikely to generate a smooth supply of ^3^He in the future. Consequently, we are now obliged to search alternatives to circumvent this ^3^He crisis.

Fortunately, low-temperature physicists already developed a ^3^He-free system, the adiabatic demagnetization refrigerator (ADR). This device makes use of magnetocaloric materials (MCMs) which are essentially paramagnetic compounds^5^. The term *magnetocaloric* was reported for the first time by Weiss and Piccard^6^, and the magnetocaloric effect (MCE) has been defined as reversible adiabatic variation in temperature of a material by an applied magnetic field. Compared with cumbersome gas-handling for ^3^He containing fridges, ADRs provide much easier operation: simple ramping-up and -down a magnet while operating (opening) a heat-switch cools down the system (see later). But further development of ADRs was delayed due to widespread adoption of the dilution refrigerator since the 1970s, because of its specific continuous cooling capacity. Although continuous cooling is also available in ADR since the initiation of space projects requiring zero-gravity cryostats^7^,^8^, most popular MCMs at low temperatures are still the paramagnetic salts which were independently proposed by Debye and Giauque^9^,^10^,^11^ 80 years ago. Until recently, the MCE was best known for the working principle of ADR but it has also yielded invaluable information about magnetic phase transitions^12^, quantum criticality^13^,^14^,^15^ and alternative refrigeration techniques^16^,^17^ in modern science.

An ideal MCM for low-temperature applications should be made of magnetic atoms, which are in a degenerate paramagnetic state down to the lowest possible temperature in zero magnetic field. Then, it should have a large volumetric entropy capacity for a good cooling power. In addition, it is preferred to be metallic and non-superconducting for better conduction of heat and easy machining. And finally, it should not degrade over time. So far, most of the materials used for temperatures below 2 K have only fulfilled the first two requirements^7^, but sophisticated treatments are mandatory to meet the standard of operation: To protect the MCMs and for a sufficient thermal contact, those non-metallic MCMs must be prepared inside a canister with special metallic structures^18^,^19^.

In this article, we demonstrate that the metallic compound YbPt_2_Sn, which satisfies all aforementioned prerequisites for a MCM at low temperatures. We have measured the specific heat capacity *C*(*T*,*B*) of polycrystalline YbPt_2_Sn^20^ down to a temperature *T*=0.05 K and magnetic field *B* up to 7 T by using a compensated heat-pulse method^21^. The entropy *S*(*T*,*B*) converted from *C*(*T*,*B*) is used to estimate the MCE and the minimum temperature accessible by adiabatic demagnetization, which results to be <0.2 K. Finally, we show a direct measurement of the MCE performed in a home-made ADR with an YbPt_2_Sn ingot and demonstrate how an ADR made with YbPt_2_Sn is a feasible and durable alternative to ^3^He-cryostats.

## Results

### Specific heat of YbPt_2_Sn

**Figure 1:**
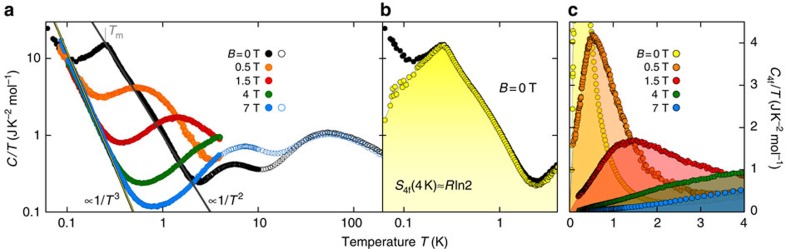
Specific heat of YbPt_2_Sn. (**a**) *T*-dependence of the specific heat capacity of YbPt_2_Sn divided by the temperature, *C*/*T*, at different magnetic fields in a double-logarithmic plot. Empty and filled symbols are data obtained with a standard ^4^He-cryostat and a dilution refrigerator, respectively. The straight lines emphasize the 1/*T*^2^ increase of *C*/*T* due to strong fluctuations and the typical 1/*T*^3^ dependence of *C*/*T* for the high-temperature side of the nuclear Schottky peak. *T*_m_=0.25 K marks the onset of the short-range magnetic ordering. (**b**) 4*f*-electron contribution *C*_4*f*_/*T* (yellow symbols) to the total specific heat *C*/*T* (black symbols) at *B*=0 after having subtracted the conduction-electron contribution *γ*=0.03 J K^−2^ mol^−1^ and the nuclear *α*_n_/*T*^3^ contribution with *α*_n_=5.8 mJ K mol^−1^ (see Supplementary Fig. 1 and Supplementary Note 1). The yellow area under the *C*_4*f*_/*T* versus *T* curve is the entropy released up to 4 K, S_4*f*_(4*K*)≈*R*ln2, the maximum entropy for a doublet ground state. (**c**) *C*_4*f*_/*T* versus *T* at *B*=0, 0.5, 1.5, 4 and 7 T.

The relevant actors in our study are the Yb^3+^ ions, which are in the magnetic 3+ valence state with total angular momentum *J*=7/2. They are exposed to a crystalline electric field (CEF) with hexagonal symmetry, which lifts the degenerate 4*f*-shell energy levels into four doublets leaving a Kramers doublet as ground state with effective spin 1/2 and maximum entropy *R*ln2*=*5.76 J K^−1^ mol^−1^ (with *R* the gas constant)^20^. This is best shown in Fig. 1a, where the specific heat capacity *C*(*T*,*B*) of YbPt_2_Sn is plotted over a broad range of temperatures 0.06≤*T*≤200 K and at selected magnetic fields 0≤*B*≤7 T. We start to look at *C*/*T* in zero field (black symbols). At high temperatures, *C*/*T* displays two broad maxima, at about 50 and 6 K, which are due to phonons and a Schottky peak caused by thermal occupation of the first excited CEF level, respectively. Below 3 K, *C*/*T* increases steeply with a 1/*T*^2^ dependence which is attributed to the emergence of short-range (intersite) magnetic correlations, which are not sufficient to induce long-range magnetic ordering^20^. Eventually, at *T*_m_=0.25 K short-range magnetic ordering sets in and is manifested as a kink in *C*(*T*)/*T*, similar to the parent compound YbPd_2_Sn^22^. Such a low *T*_m_ is very uncommon in Yb-based intermetallic systems with no sizeable Kondo interaction (like YbPt_2_Sn), which usually have transition temperatures of a few Kelvin. The nature of the magnetic ordering and the reason for the very weak magnetic exchange (≈0.8 K (ref. 20)) are still unknown, but this unique behaviour allows us to use the full unquenched entropy between *T*_m_ and 4 K for AD cooling in the same *T*-range of ^3^He systems.

At *T*<*T*_m_, *C*/*T* increases again as 1/*T*^3^ because of the nuclear contribution *C*_n_ to the specific heat from the nuclear moment of Yb, see Supplementary Fig. 1 and Supplementary Note 1. This is emphasized by the measurements at different fields where the 1/*T*^3^ increase does not change much with *B* but becomes more visible. In fact, increasing *B* rapidly suppresses the phase transition at *T*_m_. The kink at *T*_m_ transforms into a Schottky peak, which shifts to higher *T*, uncovering the large increase of *C*_n_/*T* below 1 K. This Schottky peak is the result of the Zeeman splitting of the ground state doublet and is the fundamental effect needed for the MCE.

To demonstrate this we have subtracted from all *C*(*T*)/*T* curves the small conduction-electron contribution *C*_e_/*T*(*T*→0)=*γ* and the nuclear contribution *C*_n_/*T*=*α*_n_/*T*^3^ to obtain the pure 4*f*-electron specific heat *C*_4*f*_ (see Supplementary Fig. 1 and Supplementary Note 1). In this temperature range, the phonon contribution is negligible. For *B*=0 the results are shown in Fig. 1b with *γ*=0.03 J K^−2^ mol^−1^ and *α*_n_=5.8 mJ K mol^−1^ (yellow symbols). Now, evaluating the 4*f*-electron entropy *S*_4*f*_ from the integral of *C*_4*f*_/*T* between 0.06 and 4 K (*cf.* yellow area in Fig. 1b), we obtain exactly *R*ln2, the maximum entropy for a doublet ground state. Increasing the field results in a shift of this entropy to higher *T*, as shown in Fig. 1c (coloured areas), inducing the MCE we are after.

### Entropy and MCE

**Figure 2:**
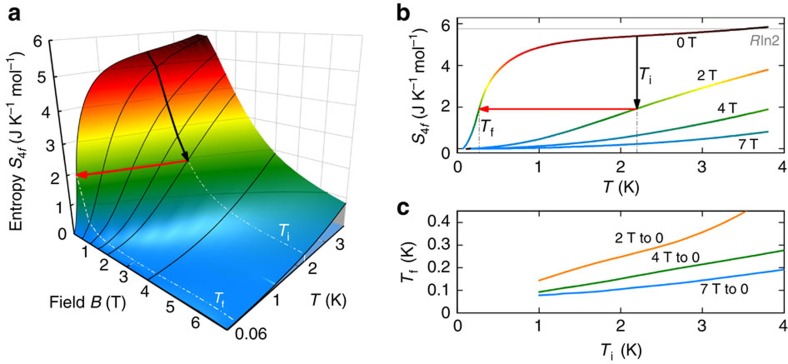
Magnetic entropy and magnetocaloric effect of YbPt_2_Sn. (**a**) Colour map of the 4*f*-electron magnetic entropy, *S*_4*f*_(*T*,*B*)=∫(*C*_4*f*_/*T*)*dT*, of YbPt_2_Sn. Black solid lines are calculated from the measured *C*_4*f*_(*T*,*B*)/*T* and the coloured surface is an interpolation of the data. Regions with the same colour are isentropic. The black arrow designates the isothermal suppression of the entropy, and the red arrow designates the adiabatic demagnetization revealing a clear MCE. (**b**) Projection into the *S*–*T* plane of the data. The grey line marks *R*ln2, which is the saturation entropy of the ground state doublet. (**c**) Initial temperature *T*_i_ dependence of the final temperature *T*_f_ for different adiabatic traces or isentropic contours.

By integrating *C*_4*f*_/*T* for all fields we can extract the full 4*f*-electron entropy *S*_4*f*_(*T*,*B*). To investigate useful adiabatic paths, we have generated a surface plot interpolating the *S*_4*f*_(*T*,*B*) curves, which is shown in Fig. 2a. As a result, the coloured surface clearly exhibits a notable MCE: The isothermal suppression of entropy (black arrow) followed by the isentropic trace (red arrow) allows to decrease the temperature. *T*_i_ and *T*_f_ designate the initial and the final temperature. The same is illustrated in Fig. 2b, that is, the projection of the data into the *S*–*T* plane. In Fig. 2c, we have plotted the expected *T*_f_ of YbPt_2_Sn as a function of *T*_i_ marked in Fig. 2a. The lower *T*_i_ the lower *T*_f_. *T*_f_ is further decreased as we suppress the entropy with stronger field. It is clear that this material can always be cooled down below 0.2 K, in a reasonable range of *T* and *B* for a standard ^4^He-cryostat and a superconducting magnet. This is because the transition at *T*_m_ does not quench much of the entropy and the MCE allows to cool down the system below *T*_m_. However, while theory suggests that it should be possible to cool down well-below 0.2 K, in real experiments (see below and Supplementary Fig. 3), only temperatures slightly below 0.2 K were achieved.

**Figure 3:**
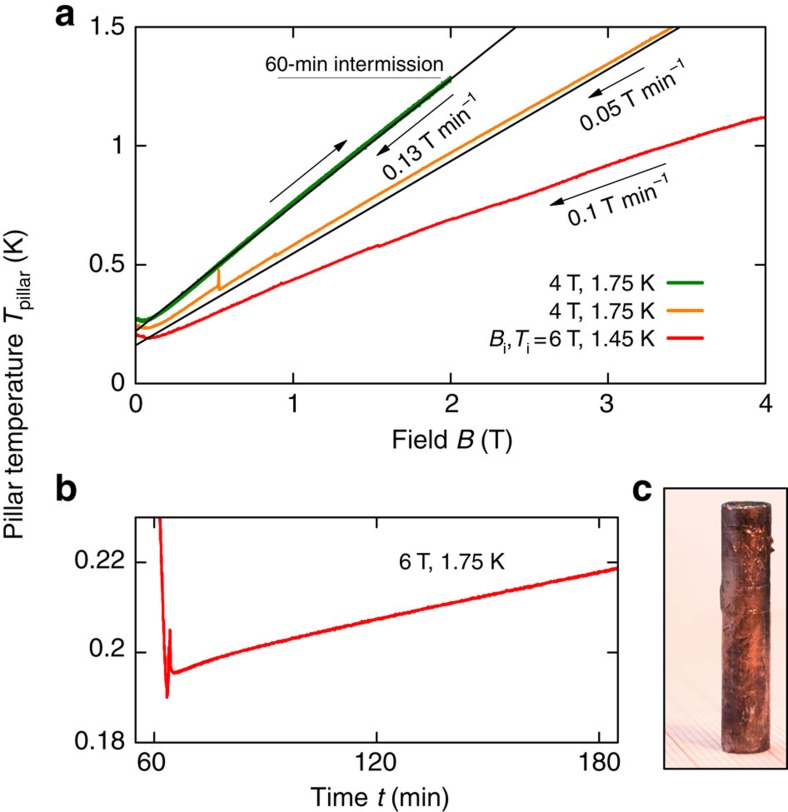
Realization of AD cooling with YbPt_2_Sn. (**a**) Measurements of the MCE by means of quasi-adiabatic demagnetization. The temperature of the YbPt_2_Sn ingot pillar (photograph), *T*_pillar_, is shown for various paths. Current record of the lowest temperature is 0.19 K, which was reached starting from 6 T and 1.45 K. From 4 T and 1.75 K, 0.22 K was reached and few hours later the temperature rose to 0.26 K. From this point, the pillar was magnetized up to 2 T and held for 1 h before it was demagnetized again. Arrows alongside each measurements indicate the directions of the sweeps and the sweep rates are also noted nearby. Almost a linear behaviour of the measured *T*_pillar_(*B*) emphasized by the straight lines is evidence of the paramagnetic MCE. (**b**) Increasing of *T*_pillar_ with time: about 0.01 K h^−1^. (**c**) The ingot pillar (10 g) of YbPt_2_Sn.

There are several materials, like paramagnetic salts or magnetic garnets (see Table 1), which are used for AD cooling in the same temperature range, but the advantage of YbPt_2_Sn is that it is a good metal and it can easily be cast into rods, making preparation of cooling pills quite simple, in contrast to salts or garnets. To test our material, we built a simple home-made ADR with a 10 g ingot pillar of YbPt_2_Sn (shown in Fig. 3c). More details will be found in Methods: ADR set-ups. Direct measurements of the MCE with the YbPt_2_Sn ingot are shown in Fig. 3a. Along the path from 6 T (red line), starting at 1.45 K, with a sweep rate of 0.1 T min^−1^, the temperature of the pillar, *T*_pillar_, had reached 0.19 K, which is about 0.1 K higher than the *T*_f_ estimated from Fig. 2. After that, the pillar began to warm up due to inevitable heat loads from the electrical wiring and supporting structures (*cf.* Fig. 3b). The magnet power supply was the source of oscillations and occasional spikes in *T*_pillar_(*B*). Another adiabatic path from 4 T, starting from 1.75 K with higher sweep rate of 0.13 T min^−1^ ended at 0.22 K, which is also 0.1 K higher than the estimated *T*_f_. The uppermost cycle shows a ramping-up from 0 T and 0.26 K to 2 T and 1.3 K, and a successive ramping-down to 0 T and 0.27 K after 1 h of intermission. Also here, the 0.27 K achieved by MCE from 2 T and 1.3 K is about 0.1 K higher than the estimation. The discrepancy between *T*_pillar_(0) and *T*_f_ is reasonable given that thermal and mechanical insulations were not as good as in a commercial standard ADR. In fact, the Kapton tubes guarantee good thermal insulation only below 1 K. We have also tested YbPt_2_Sn in a commercial physical properties measurement system (PPMS) (Quantum Design) by using an even simpler design without a heat-switch (see Supplementary Figs 2 and 3) and reached about 0.16 K. However, our experimental set-up provided a good enough thermal insulation at the lowest temperatures, which is reflected in a warming-up rate of 0.01 K h^−1^ at about 0.2 K, as shown in Fig. 3b. The reversibility and linearity of *T*_pillar_(*B*), regardless of the sweep rate, indicates negligible eddy-current heating (see green curves in Fig. 3a). Consequently, the MCE observed in YbPt_2_Sn is reminiscent of that of an ideal paramagnet. An ideal paramagnet is universally described by (∂*T*/∂*B*)_*S*_=(*T*/*B*) and *T*_f_=*T*_i_(*B*_f_/*B*_i_) regardless of material parameters, where *B*_i_ and *B*_f_ are the initial and final magnetic fields along an adiabatic path, respectively. This means that at *B*_f_=0, *T*_f_=0. This is not the case for YbPt_2_Sn where we observe a finite *T*_f_=*T*_pillar_(0)≈0.2 K at *B*_f_=0 (see black straight lines in Fig. 3a). This is certainly due to the magnetic weak exchange interaction, which is of the same order of magnitute of *T*_pillar_(0) and *T*_m_ (ref. 20).

## Discussion

How good is YbPt_2_Sn compared with standard MCM materials for the same *T*-range? In a MCM, a weak magnetic interaction is beneficial to achieve a low base temperature. Moreover, the spin *J*_GS_ of the ground state determines the saturation entropy *R*ln(2*J*_GS_+1) and, therefore, a large *J*_GS_ is advantageous for a stronger cooling power. It is worth mentioning that for practical purposes it is not the molar entropy but it is the entropy density of the material which is the relevant quantity. Finally, a large *g*-factor is desirable because the suppression of entropy by a magnetic field becomes more susceptible. Hence, for a MCM large *J*_GS_, *g*, density *d* and a weak magnetic interaction are desirable, but large values of the first three parameters promote a strong magnetic interaction. Thus creating a low-temperature magnet with a weak interaction usually is in conflict with having a large cooling capacity and *vice versa*. Table 1 shows relevant parameters for paramagnetic salts and garnets, which are popular MCMs for ADR <1 K. In chromium potassium alum and ferric ammonium alum, first order magnetic phase transitions appear near *T*_m_≈10 mK (ref. 23) and *T*_m_≈30 mK (ref. 24), respectively. Normally, the ordering temperature *T*_m_ is indicative of the lowest possible temperature as entropy is quenched with a phase transition. As mentioned above, the intuitive approach to keep large distance between magnetic ions to obtain low *T*_m_ (typical of these materials) is accompanied by the downside of having low volumetric entropy capacity, *S*_*v*_. For gadolinium gallium garnet^25^ and dysprosium gallium garnet^26^, one can have much higher *S*_*v*_ than salts at the expense of *T*_m_. Moreover, it is common to have higher *T*_m_ as *J*_GS_ is increased^27^. In comparison to salts, YbPt_2_Sn has more than eight times larger *d*, and roughly three times larger *S*_*v*_ while it orders at 0.25 K. At first glance, the tendency to relinquish a low *T*_m_ over *S*_*v*_ seems to be obeyed. However, in contrast to rare-earth garnets and other rare-earth MCMs^28^, the magnetic ordering around 0.2 K cannot be accounted for by neither *J*_GS_ nor *d* dependence. Most of all, the weak and continuous magnetic ordering makes YbPt_2_Sn unique because it can be practically cooled below *T*_m_. It is also worth noting that similar compounds like YbPd_2_Sn (with *T*_m_=0.23 K (refs 22, 29)) and YbPt_2_In (with *T*_m_=0.18 K (ref. 20)) all show doublet ground states and weak ordering, but YbPd_2_Sn becomes superconducting at 2.3 K (that is, a bad thermal conductor) and YbPt_2_In has not yet been tested. It is worth mentioning that there are materials which show maximal MCE at the verge of metamagnetic transitions^30^. However, those materials require a demagnetization towards a nonzero field and the small entropy change would provide a weaker cooling effect when compared with that of YbPt_2_Sn.

The aforementioned exceptional properties of YbPt_2_Sn allowed us to build an ADR with a metallic MCM operable below the base temperature of a ^3^He-cryostat. This material can be easily cast into various shapes and directly attached to the cooling target without any complications. It is a good metal and no additional metallic structures are needed for the thermal conduction unlike in paramagnetic salts or garnets, which are very poor thermal conductors <1 K. In this sense, while the physical origin of the weak interaction still needs to be clarified, it is an important feature that makes YbPt_2_Sn a viable new metallic refrigerant material for ADR. YbPt_2_Sn makes ADR more accessible by resolving complications of preparing non-metallic materials, which have been used since the 1930s. At the same time, it provides low enough base temperatures so that it can be chosen as an alternative to ^3^He-cryostats. We hope that our discovery will accelerate low-temperature physicists to escape from the ongoing ^3^He crisis.

## Methods

### Material preparation

Despite the high vapour pressure of divalent Yb metal, YbPt_2_Sn can easily be prepared in a standard arc-melting furnace by first reacting Yb with the low melting Sn and then adding Pt. Details are given in ref. 20. The pillar used for the PPMS set-up (see Supplementary Fig. 2) and the rod for the home-made ADR were obtained by casting pre-reacted YbPt_2_Sn in an appropriate mould in an arc-melting furnace and in a commercial high-frequency casting system (see Supplementary Movie 1), respectively.

### Heat capacity

Measurements of heat capacity have been carried out in a dilution refrigerator (Oxford Instruments) for temperatures 0.05≤*T*≤4 K and magnetic field *B* up to 7 T by using a compensated heat-pulse method described in ref. 21. For *T*>2 K, the measurements were performed in a 7 T PPMS.

### ADR set-ups

To test the cooling capability and MCE of YbPt_2_Sn, we have built two different refrigeration systems. One follows the standard structure of commercial ADR, which is equipped with a mechanical heat-switch, and the other is a simple miniaturized version without heat-switch that can be used in a commercial PPMS, as shown in Supplementary Fig. 2. In the former set-up, we have modified a top-loading 1 K pot cryostat. The sample stage (thin brass disc) is fixed below the 1 K pot by using a pair of Kapton straws and is located at the stray-field compensation zone of the superconducting magnet. Beneath this stage, two thin brass rods are stretched to the magnet centre from opposite edges of the sample stage, and the end of each rod is joined perpendicular to each arms of ‘Φ'-shaped brass part. The mass of the whole brass structure is about 30 g. The YbPt_2_Sn ingot pillar shown in Fig. 3c is clamped at the centre of this thin metal piece. A push-pull manual actuator is used to activate the mechanical heat-switch. Further details and specifications of this set-up will be reported elsewhere.

## Additional information

**How to cite this article:** Jang, D. *et al*. Large magnetocaloric effect and adiabatic demagnetization refrigeration with YbPt_2_Sn. *Nat. Commun.* 6:8680 doi: 10.1038/ncomms9680 (2015).

## Supplementary Material

Supplementary Information Supplementary Figures 1-3, Supplementary Notes 1-3 and Supplementary References

Supplementary Movie 1 Preparation of the metallic magnetocaloric material YbPt_2_Sn. The new magnetocaloric material YbPt_2_Sn is cast into a rod in a cold crucible system. A strong radio frequency (RF) magnetic field induces large electrical currents in pre-reacted YbPt_2_Sn pieces which heat them up and let them melt, while the water-cooled copper crucible stays cold. Turning of the RF generator lets the melt drop into a cylindrical mold underneath the crucible.

## Figures and Tables

**Table 1:** Comparison of parameters for various magnetocaloric materials.

	***J***_**GS**_	***g***	***T***_**m**_ **(mK)**	***d*** **(g** **cm**^−3^**)**	***S***_***v***_ **(J** **K**^−1^ **cm**^−3^**)**
CPA^23^	3/2	2	10	1.83	0.042
FAA^24^	5/2	2	30	1.71	0.052
GGG^25^	7/2	2	800	7.10	0.363
DGG^7^,^26^	1/2	−*	400	7.30	0.123
YbPt_2_Sn	1/2	5.6	250	14.6	0.127

CPA, chromium potassium alum—CrK(SO_4_)_2_*·*12(H_2_O); DGG, dysprosium gallium garnet—Dy_3_Ga_5_O_12_; FAA, ferric amonium alum—FeNH_4_(SO_4_)_2_*·*12(H_2_O); GGG, gadolinium gallium garnet—Gd_3_Ga_5_O_12_.

^*^DGG has strong anisotropy: *g*_*x*_=10.72, *g*_*y*_=1.54 and *g*_*z*_=8.52 (ref. 27).

*J*_GS_, *g*, *d* and *T*_m_ are the effective spin quantum number of the ground state, the *g*-factor, the material density and the temperature of the magnetic ordering, respectively. *S*_*v*_ is the volumetric entropy capacity obtained by *R*ln(2*J*_GS_+1) × (*d*/*M*), where *M* is the molar mass per magnetic ion.
