# Supplementary material for: Large magnetocaloric effect and adiabatic demagnetization refrigeration with YbPt2Sn
Source: Nat Commun. 2015 Oct 23;6:8680. doi: 10.1038/ncomms9680 (PMC4846311; doi:10.1038/ncomms9680)
Supplement: Supplementary Information — Supplementary Figures 1-3, Supplementary Notes 1-3 and Supplementary References [file ncomms9680-s1.pdf]

Supplementary Figures

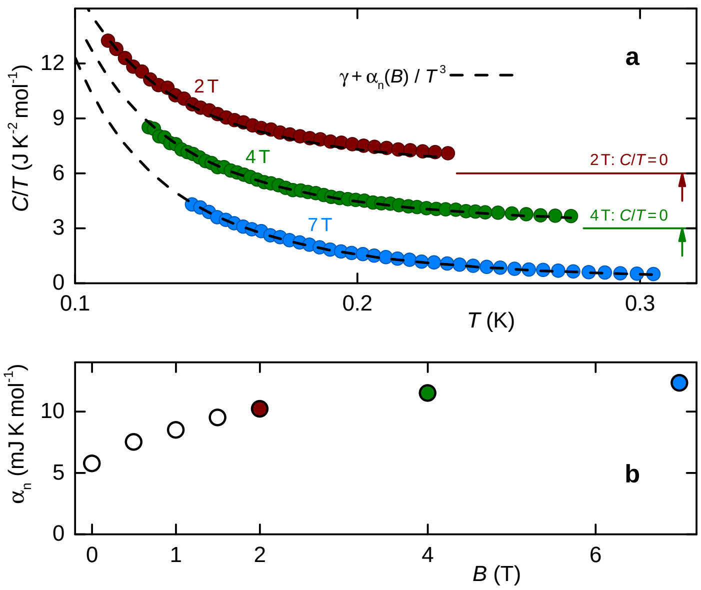

Supplementary Figure 1: **Nuclear specific heat.** **a**, Low-temperature part of C/T at high magnetic fields where the main contribution to $C(T)$ is the high-T tail of the nuclear Schottky peak $C_{\mathrm{n}} \propto 1/T^2$. Dashed lines are fits with $C_{\mathrm{n}}/T = \gamma + \alpha_{\mathrm{n}}/T^3$. The data and fits are shifted by about 3 and 6 J K^{-2} mol^{-1} for 4 and 2 T, respectively. The conduction electron specific heat $C_{\mathrm{e}}/T = \gamma = 0.03\,\mathrm{J}\ \mathrm{K}^{-2}\ \mathrm{mol}^{-1}$ is constant for all fields. **b**, Field dependence of the fitting parameter α_{n}.

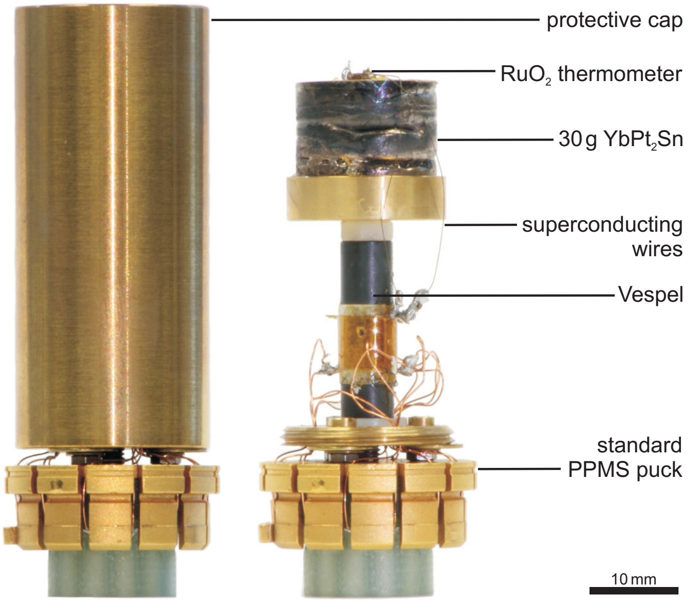

Supplementary Figure 2: **Set-up for the PPMS.** The set-up is made up of a PPMS puck, a vespel tube, a brass plate which holds a single ingot pillar (30 g) of $YbPt_2Sn$. The protective cap can be screwed on the puck.

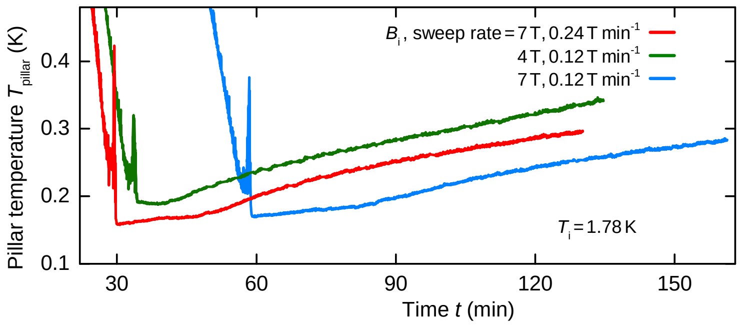

Supplementary Figure 3: **AD cooling by $YbPt_2Sn$ in the PPMS set-up.** Three selected measurement runs with the PPMS set-up. The temperature of the RuO_2 thermometer on the $YbPt_2Sn$ pillar is plotted over the time. The lowest temperature was achieved with a sweep rate of 0.24 T min^{-1} starting from 7 T and about 1.8 K (red curve).

Supplementary Notes

Supplementary Note 1

Nuclear specific heat: The specific heat of $YbPt_2Sn$ shows a pronounced increase at temperatures below 1 K (see Fig. 1a of the main text). This is due to the the splitting of the nuclear energy levels of the Yb ions because of their quadrupole moment and the Zeeman energy produced by the hyperfine field. Magnetic rare earth elements usually present a large, easily polarisable magnetic $4f$ moment and a huge hyperfine coupling between this moment and the nuclei. Therefore, the nuclear specific heat in rare earth based systems is generally completely dominated by the contribution of the rare earth elements. This is the case for $YbPt_2Sn$, too, as confirmed by an evaluation of the relevant parameters [1, 2]. Yb exists in a few stable isotopes, but only ^{171}Yb and ^{173}Yb have a nuclear moment ($I_{171} = 1/2$ and $I_{173} = 5/2$). In addition, ^{173}Yb has a quadrupole moment of 2.8×10^{-28}m^2.

Considering that below 1 K the phonon contribution to the specific heat is negligible, the conduction-electron and nuclear specific heats, $C_\mathrm{e} + C_\mathrm{n}$, can be approximated by the Sommerfeld coefficient γ times T and the asymptotic α_n/T^2 power law of the high-T Schottky peak, respectively. In Supplementary Figure 1a, the measured specific heat $C(T)$ is plotted as C/T vs T at 2, 4 and 7 T. We have fitted those curves with the previous formula (black dashed lines). The fit is excellent and provides an almost constant Sommerfeld coefficient $\gamma = 0.03$ J K^{-2} mol^{-1} for all curves and different values for α_n which is field dependent, as expected. At smaller fields, the direct fitting is not reliable because the contribution from the $4f$ electrons and we kept γ constant. Supplementary Figure 1b shows the field dependence of α_n. We first note that in such magnetic Yb systems the Yb hyperfine field is completely dominated by the polarisation of the $4f$ shell, and is thus proportional to the local static $4f$ moment. The direct effect of an external field is not relevant. At $B = 0$, the finite and large value of $\alpha_\mathrm{n} = 5.8$ mJ K mol^{-1} is likely mostly due to the local static $4f$ moment in the short-range ordered magnetic state. Applying a magnetic field increases slightly the size of the local static moment and thus result in a small increase of α_n. The size of the nuclear contribution at $B = 7$ T agrees with that expected for a fully polarised Yb moment.

Supplementary Note 2

Set-up for the PPMS: The fact that $YbPt_2Sn$ is metallic and easy to machine gave us the idea to built a simple set-up for the PPMS in order to measure resistivity below 2 K. Our set-up is shown in Supplementary Figure 2 and it has been inspired by that of Dai Aoki's group (CEA) in Grenoble. The lower part consists of the standard PPMS puck that can be attached to the bottom of the PPMS and set at the base temperature of 1.8 K or slightly lower. On the top of the puck we have attached a small vespel tube for thermal isolation. At the very top we have connected a brass plate and a 30 g sample of $YbPt_2Sn$. To measure the temperature we have used a calibrated RuO_2 thermometer connected with single filaments of superconducting wires. The whole structure is closed by a brass cylinder (protective cap) which remains at 1.8 K (left side of Supplementary Figure 2) and is needed to prevent thermal radiation heating from the top of the PPMS. Even though the $YbPt_2Sn$ sample is positioned at a few centimeters above the puck, the magnetic field at the sample is still very close to that at the puck.

For every single cool-down we followed the same procedure: The magnetic field was set at the desired value, for instance, 7 T, then exchange gas was inserted in the PPMS (about 0.1 Torr) and the system was let to cool down to about 1.8 K. After that, we pumped out the exchange gas reaching high vacuum (about 0.1 mTorr) and swept the field down to zero. Considering that eddy currents warm up the sample we have tried with different sweep rates. Our best runs are shown in Supplementary Figure 3 where we have plotted the temperature of the RuO_2 thermometer as a function of the time. In all runs, starting from 7 or even 4 T at about 1.8 K we were able to achieve temperatures below 0.2 K. In particular, in the run from 7 T with 0.24 T min^{-1} we have reached 0.16 K (red curve). After having reached the minimum temperature at $B = 0$, the temperature increased with a rate of about 5×10^{-3} K min^{-1} due to the non-ideal thermal isolation. However, this rate is slow enough to perform precise resistivity measurements.

With this experiment we want to show how easy it is to build an ADR by using $YbPt_2Sn$ and that even in commercial systems this material can be used to achieve temperatures below 0.2 K.

Supplementary Note 3

Eddy currents: Since $YbPt_2Sn$ is metallic, eddy currents cause heating effects. We have shown in the main text that these effects are negligible in $YbPt_2Sn$. To support the experimental evidence we calculate here with a simplified model the temperature change ΔT at 0.1 K caused by sweeping the magnetic field from 4 T to zero at a rate of 0.1 T min^{-1} on the 10 g pillar displayed in Fig. 3c of the main text. We consider this pillar to be a cylinder

of radius $R = 3\,\text{mm}$, high $l = 30\,\text{mm}$ and molar mass $m_{\text{mol}} = 681.9\,\text{g}\,\text{mol}^{-1}$. Since $YbPt_2Sn$ is a paramagnet with a susceptibility $\chi < 1$ and for a cylinder with $R/l = 10$ the demagnetisation factor is about 0.02, the internal field is approximately equal to the external field. The component of the electric field $\mathbf{E}$ (in $\hat{\boldsymbol{\phi}}$ direction) induced by the external magnetic field $\mathbf{B}(t)$ (oriented along the $\hat{\mathbf{z}}$ axis) is $E_\phi = -r(\partial B_z/\partial t)/2$. If we assume temperature- and field-independent resistivity, ρ, and after integrating the infinitesimal heat loss at arbitrary position r, the power loss per unit volume is $P/V = E_\phi^2/2\rho = R^2(\partial B_z/\partial t)^2/8\rho$. We take here the residual resistivity of $YbPt_2Sn$, $\rho = 250\,\mu\Omega\text{cm}$ (RRR ≈ 2), from Ref. 3. With $\partial B_0/\partial t = 0.1\,\text{T min}^{-1}$ we obtain $P/V = 12.5 \times 10^{-7}\,\text{W m}^{-3}$ and, considering the volume of the pillar $V = 8.5\times10^{-7}\,\text{m}^3$, the total dissipated power is $P \approx 106\times10^{-14}\,\text{W}$. Sweeping the field from 4 T to zero with $0.1\,\text{T min}^{-1}$ takes $t = 40\,\text{min}$ and the heat produced is $Q = P\cdot t \approx 2.5\,\text{nJ}$. At 0.1 K, $C/T \approx 10\,\text{J K}^{-2}\,\text{mol}^{-1}$ which results in a specific heat for the whole 10 g of $YbPt_2Sn$ of $C = 14.7\,\text{mJ K}^{-1}$. Finally, $\Delta T = Q/C \approx 1.7\times10^{-7}\,\text{K}$ $\approx 20\,\mu\text{K}$, which is definitely negligible at 0.1 K.

Supplementary References

[1] Bleaney, B. Hyperfine Interactions in Rare-Earth Metals. *Journal of Applied Physics* **34**, 1024–1031 (1963).

[2] Steppke, A. *et al.* Nuclear contribution to the specific heat of $Yb(Rh_{0.93}Co_{0.07})_2Si_2$. *Phys. Status Solidi (b)* **247**, 737–739 (2010).

[3] Gruner, T. *et al.* Unusual weak magnetic exchange in two different structure types: $YbPt_2Sn$ and $YbPt_2In$. *Journal of Physics: Condensed Matter* **26**, 485002 (2014).
